# High-Gain Omnidirectional Dual-Polarized Antenna for Sink Nodes in Wireless Sensor Networks

**DOI:** 10.3390/s22030788

**Published:** 2022-01-20

**Authors:** Yongjian Zhang, Yue Li

**Affiliations:** 1 Department of Electronic Engineering, Tsinghua University, Beijing 100084, China; zhangyj18@mails.tsinghua.edu.cn; 2 Beijing National Research Center for Information Science and Technology, Tsinghua University, Beijing 100084, China

**Keywords:** high-gain antenna, omnidirectional radiation patterns, dual-polarized antennas, sink nodes, wireless sensor networks

## Abstract

In wireless sensor networks (WSN), a sink node receives signals from a large number of sensor nodes. Hence, the sink nodes are required to integrate compact antennas with high performances, such as high gain, dual polarizations, and omnidirectional radiation. In this paper, a high-gain omnidirectional dual-polarized (HGODP) antenna with a slot-cavity structure is proposed for WSN. The proposed antenna integrates dual omnidirectional antennas with orthogonal polarizations, i.e., a thin open-ended cavity for horizontal polarization and four folded slots for vertical polarization. Due to the orthogonal operating modes of the dual polarizations, the antenna configuration is constructed within a compact volume, but with an independent design. A prototype of the proposed antenna is fabricated and measured within a ruler-like profile. The experimental results show that the realized gains are higher than 6.5 dBi and are achieved for both dual polarizations in 2.37~2.54 GHz. With the merits of high gain, high isolation, and omnidirectional radiation, the proposed compact antenna exhibits promising usage for sink nodes in WSN.

## 1. Introduction

The dramatically booming requirement of wireless sensor networks (WSN) demand more sensor nodes with sensing ability for different applications, such as ecological monitoring, health surveillance, and traffic control [1,2,3,4]. To ensure the stability of a reception link from plenty of the sensor nodes, high-performance antennas with high gains, orthogonal polarizations, and omnidirectional coverage are required in sink nodes. First, high-gain antennas allow a longer wireless transmission distance between the sink node and sensor nodes [5,6,7]. Second, dual-polarized antennas have the capabilities of avoiding the polarization mismatch and reducing the system volume compared with a pair of spatially-separated single-polarized antennas [8,9,10]. Third, omnidirectional antennas provide a 360° full-space radiation pattern in the azimuthal plane, leading to a broad sensor coverage and flexible system alignment [11,12,13].

For high-gain omnidirectional dual-polarized (HGODP) antenna designs, it is critical to choose radiating elements with consideration of the system dimensions, port isolation, and gain variation (the difference between the maximum and the minimum gains). Recently, HGODP antennas have been widely studied by three methods. The first one is the employment of multiple directional dual-polarized antennas in a rotationally symmetric array [14,15,16,17,18,19,20]. For example, an omnidirectional dual-polarized (ODP) antenna composed of four vertical dipoles and four horizontal dipoles is presented in [14], which suggests that the port isolation is higher than 25 dB and has a bandwidth of 30%. Reference [15] adopts an inverted-cone monopole for vertical polarization and two pairs of dipoles for horizontal polarization. Another method to realize HGODP antennas is to locate a linear array of ODP elements. Two types of ODP elements are achieved by locating two orthogonal slots carved onto a column in [21]. By carving the horizontal-polarized slots on the cavity with vertical polarization, [22,23,24] present ODP antennas with high isolation. Besides, the designs in [10] and [25] realize a high-gain radiation pattern by using an array of several separated antenna elements at a certain distance. These high-gain antennas combine the antenna elements of a high profile [21], irregular structure [22,23,24,25] or within a certain distance, which will result in high grid lobes, complicated feeding structures, or large system sizes for array design. The third potential method to realize HGODP antennas is directly integrating dual high-gain omnidirectional antennas with orthogonal polarizations. However, the integration of two single-polarized antennas with high-gain omnidirectional radiation in the azimuthal plane has not been intensively studied and presented.

**Figure 1:**
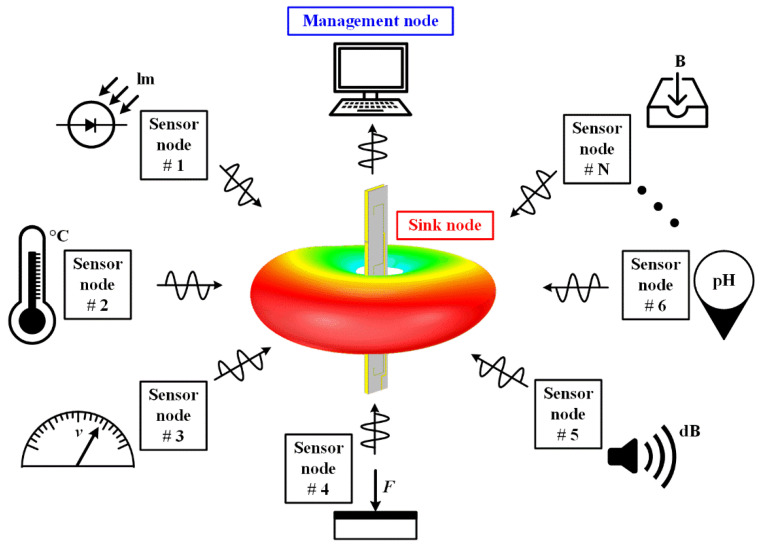
Application scenarios of the proposed high-gain omnidirectional dual-polarized (HGODP) antenna for sink nodes in wireless sensor networks (WSN).

In this work, a slot-cavity structure with a ruler-like profile is presented to integrate dual high-gain omnidirectional single-polarized antennas. As shown in Figure 1, the proposed HGODP antenna is adopted into the sink nodes and serves multiple sensor nodes in the azimuthal plane in WSN. For practical applications, the proposed antenna is located on the same horizontal plane as other sensor nodes, such as the desktop of a smart home system and the car roof of Internet of Vehicles. In this antenna, a long, open-ended cavity is fed at its zeroth-order mode along the radiating aperture for horizontal polarization. For vertical polarization, four folded slots with a proper spacing are etched onto the metallic cavity. By integrating these two structures with orthogonal polarizations, a prototype of the antenna is constructed and fabricated. The measured results show that an isolation higher than 33 dB is achieved in the band of 2.37~2.54 GHz for both dual polarizations. In addition, the realized gains are higher than 6.5 dBi and are obtained within a compact ruler-like profile with a size of 250 × 27.8 × 5 mm^3^ (2*λ*_0_ × 0.23*λ*_0_ × 0.04*λ*_0_, *λ*_0_ is the free space wavelength at 2.44 GHz). Compared with the previous HGODP antenna designs [14,15,16,17,18,19,20,21,22,23,24,25], this antenna realizes the integration of a dual high-gain omnidirectional single-polarized antenna, leading to a compact volume and high isolation. Besides, these two single-polarized antennas can be designed independently, although they are collocated together.

## 2. Antenna Design and Parametric Analysis

### 2.1. Antenna Configuration

**Figure 2:**
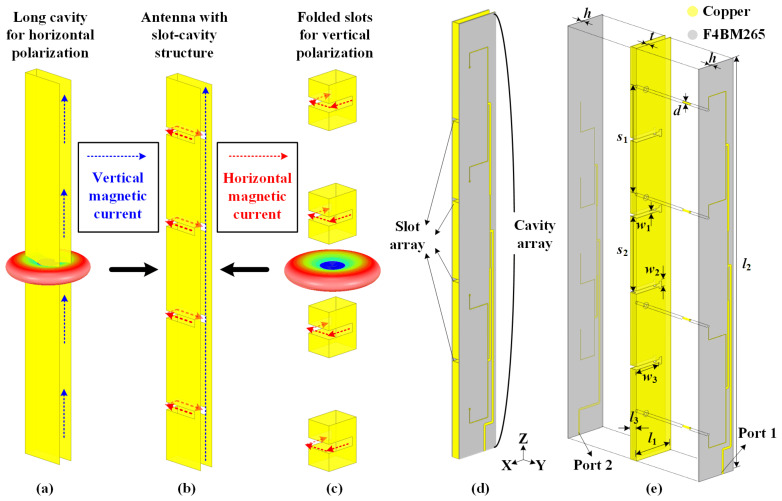
Evolution process of the proposed slot-cavity antenna with a ruler-like structure for HGODP radiation. (**a**) Long cavity for horizontal polarization. (**b**) Antenna with slot-cavity structure for dual polarizations. (**c**) Folded slots for vertical polarization. (**d**) Perspective view and (**e**) exploded view of the proposed slot-cavity antenna.

Figure 2 illustrates the evolution process of the proposed slot-cavity antenna with a ruler-like structure for HGODP radiation. The structure of the horizontal-polarized cavity is depicted in Figure 2a. When the long cavity is excited uniformly, a series of equivalent vertical magnetic currents are realized, providing high-gain omnidirectional radiation for horizontal polarization. For vertical polarization, a group of folded slots is directly carved onto the column sidewalls, as shown in Figure 2c. When the slots operate at their half-wavelength mode, several horizontal magnetic currents are equivalently obtained along the slots, causing omnidirectional vertical-polarized radiation. Based on the dual single-polarized antennas, a slot-cavity antenna is integrated within a ruler-like profile, as shown in Figure 2b. The realized horizontal and vertical magnetic currents are perpendicular with each other, leading to the orthogonal operating modes of dual polarizations. Ultimately, the proposed antenna achieves HGODP radiation and high port isolation within such a compact volume. It is worth mentioning that a configuration with a longer cavity and more slots can satisfy the requirements of higher-gain characteristics. Hence, this extendable integration method exhibits promising potentials in wireless sensor networks.

The perspective and exploded views of the proposed antenna are shown in Figure 2d,e. The yellow part represents copper, and the grey part is the F4BM265 dielectric (*ε*_r_ = 2.65 and tan *δ* = 0.002). The proposed antenna is composed of an all-metal cavity with a thickness of *l*_3_ = 5 mm and two feeding networks printed on the substrate with a thickness of *h* = 0.5 mm. The width of the metal cavity *l*_1_ is optimized at 27.8 mm (a quarter wavelength at the center frequency of 2.44 GHz), and the whole height of the cavity is 250 mm (two wavelengths at 2.44 GHz). The thickness of the cavity sidewall is *t* = 0.5 mm. A four-way power divider is adopted as the feeding network to uniformly excite the long, open-ended cavity and is connected to four identical metal probes with a diameter of *d* = 1.2 mm and a spacing *s*_1_ = 65.5 mm. When fed through Port 1 for horizontal polarization, the thin cavity operates at the TE_0.5,0,0_ mode, providing the omnidirectional radiation pattern in the azimuthal plane. For vertical-polarized radiation, four folded T-shaped slots with a width of *w*_1_ = 2 mm are directly carved onto the sidewalls of the thin cavity, with a spacing of *s*_2_ = 46 mm between two adjacent slots. The total length of each slot is *l*_3_ + *w*_3_ × 2 + *w*_2_ = 46.4 mm, which is nearly a half of the resonant wavelength at 2.44 GHz. These four slots are microstrip-fed using another similar four-way power divider feeding network on the other side. When fed through Port 2, each slot operates at the half-wavelength mode, causing the azimuthally omnidirectional radiation pattern. By combining the metallic cavity and two substrates as a sandwich, dual orthogonal-polarized antennas are integrated with a compact ruler-like structure and high port isolation. Here, all simulated results of the antenna are numerically analyzed by the commercial software, ANSYS Electronics Desktop 18.0. When one port is fed for the corresponding polarization, the other port is connected with a 50-Ω matched load.

### 2.2. Omnidirectional Radiation for Horizontal Polarization

**Figure 3:**
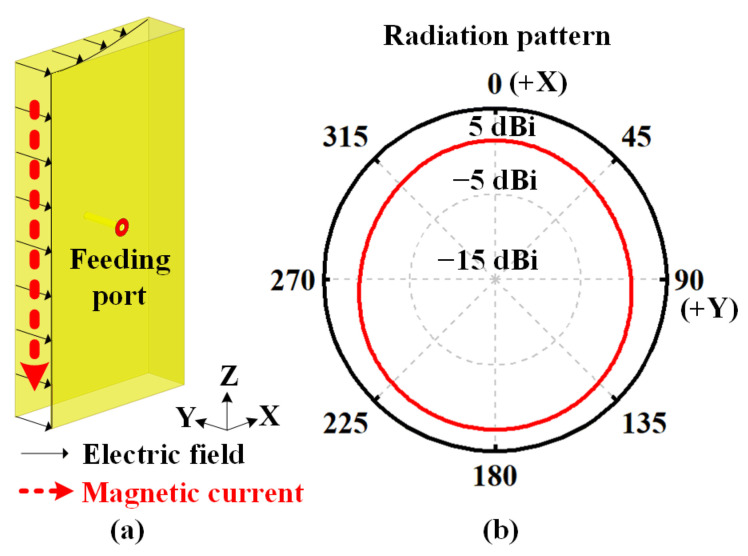
Geometry of the short cavity for horizontal polarization. (**a**) Uniform E-field distribution and equivalent magnetic current on the aperture of a cavity at the TE_0.5,0,0_ mode. (**b**) Omnidirectional radiation pattern in the azimuthal plane.

Figure 3 shows a short cavity with a height of a half wavelength for horizontal polarization. This cavity has only three metallic sidewalls. By tuning the position of the metal feeding probe, the thin cavity operates at the TE_0.5,0,0_ mode (the electric field is within the distribution of a quarter wavelength along the *X*-axis but is uniform along the *Y*-axis and *Z*-axis). The equivalent magnetic current causes the omnidirectional radiation pattern in the azimuthal plane, as illustrated in Figure 3b.

**Figure 4:**
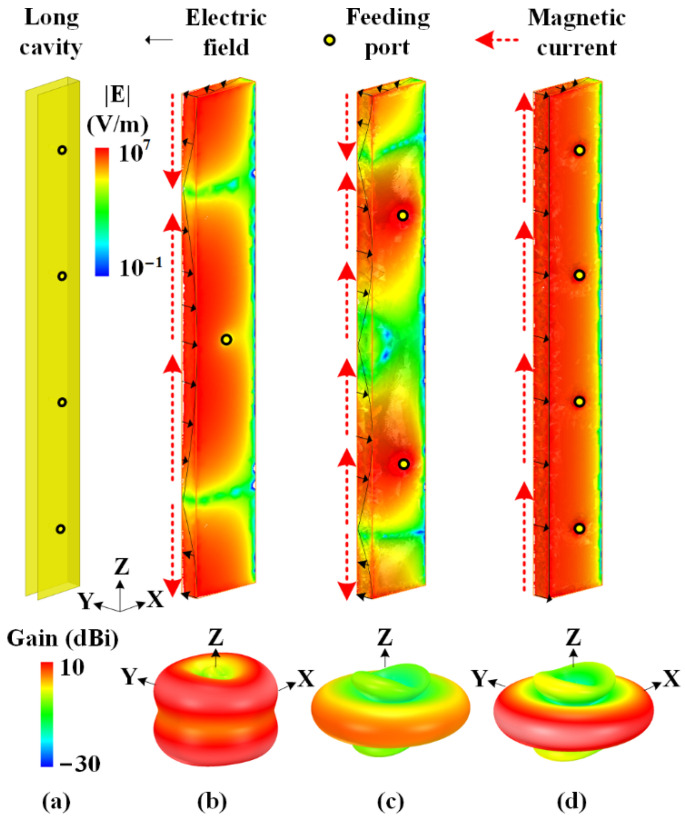
Uniform electric field distribution on the aperture of the horizontally polarized long cavity for high gain. (**a**) Proposed long cavity with the length of two wavelengths. Electric field distributions and radiation patterns of horizontally polarized cavities with (**b**) 1, (**c**) 2, (**d**) 4 feeding ports.

Figure 4 illustrates the effect of the number of feeding probes on the electric field of the long cavity. Here, the heights of three cavities are all taken as two wavelengths. When fed through one feeding port in the middle of the cavity, as shown in Figure 4b, the equivalent magnetic currents along the cavity aperture are out-of-phase and the thin cavity operates at the TE_0.5,0,2_ mode with a one-wavelength electric field distribution along *Z*-axis. Therefore, the peak gain in the three-dimensional radiation pattern is not in the azimuthal plane. In Figure 4c, two feeding ports are utilized to excite the long cavity in the same phase. This cavity operates at the TE_0.5,0,3_ mode, and the electric field is mainly in the same direction on most of the aperture, generating an omnidirectional radiation pattern. However, the direction of the electric field on the upper and lower part of the aperture is opposite to that in the middle part, reducing the peak gain in the azimuthal plane. The number of feeding ports is optimized and finally taken as four, as the field distributions and radiation properties are illustrated in Figure 4d. By exciting these four feeding ports in the same amplitude and phase, the radiating aperture of the long cavity operates at the TE_0.5,0,0_ mode. The uniform electrical field along the radiating aperture of the cavity results in a high aperture efficiency. Hence, by utilizing four feeding ports, the thin open-ended cavity with the height of two wavelengths achieves an omnidirectional radiation pattern in the azimuthal plane for horizontal polarization.

### 2.3. Omnidirectional Radiation for Vertical Polarization

**Figure 5:**
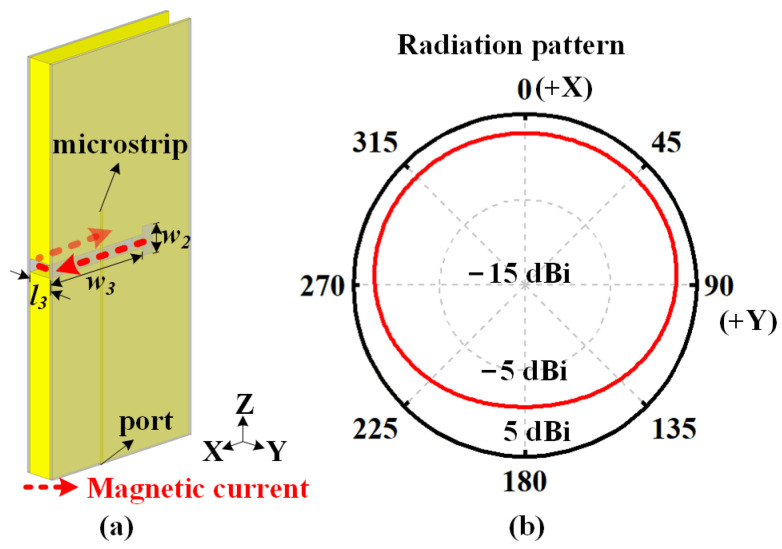
Geometry of one vertically polarized slot. (**a**) Distribution of the equivalent magnetic current along the slot at the half-wavelength mode. (**b**) Omnidirectional radiation in the azimuthal plane.

Figure 5 illustrates the configuration of the one slot for vertical polarization. A folded slot is carved into the middle of the thin cavity with a height of a half wavelength. Two dielectric substrates are attached onto the cavity sidewalls, and a microstrip line is properly fabricated onto one substrate as the feeding structure. The total length of the slot is *l*_3_ + *w*_3_ × 2 + *w*_2_ = 46.4 mm, which is nearly a half of the resonant wavelength at the center frequency of 2.44 GHz. By tuning the position and length of the microstrip line, the slot element operates at the first-order mode, causing an equivalent magnetic current along the slot and an omnidirectional radiation pattern, as shown in Figure 5b.

**Figure 6:**
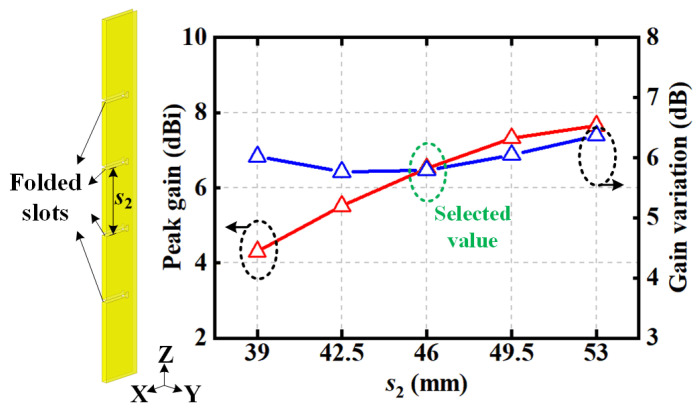
Radiation properties of vertically polarized slots in the azimuthal plane with different values of distance between adjacent slots *s*_2_.

For vertical polarization, four identically folded slots with spacing *s*_2_ are uni-formly excited and directly carved onto the cavity sidewalls, as depicted in Figure 6. The radiation properties with different values of *s*_2_ are also illustrated, including the peak realized gains and gain variations in the azimuthal plane. As spacing *s*_2_ increases, the peak gain becomes larger with a narrower main beam in the elevated plane. Due to the mutual coupling between the slots, an obvious gain variation is realized in the azimuthal plane. When the space between adjacent slots is selected as *s*_2_ = 46 mm, the active impedance match is achieved and the energy is uniformly quartered on these four slots, causing a gain variation of less than 6 dB, which is acceptable for practical applications.

### 2.4. Dual-Polarized Slot-Cavity Antenna

**Figure 7:**
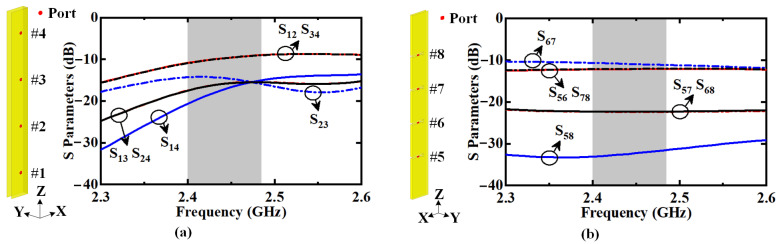
Mutual coupling between feeding ports for (**a**) horizontal and (**b**) vertical polarizations.

By integrating the long cavity and group of slots, the proposed dual-polarized antenna is built with a ruler-like profile. To excite this HGODP antenna, two compact feeding networks are designed and arranged onto the sidewalls of the cavity. Before the detailed design of the feeding networks, the mutual coupling between the feeding ports is analyzed for each polarization. Figure 7a illustrates the mutual coupling between the horizontally polarized feeding ports #1–#4. As seen, the isolations between adjacent ports, such as S_12_ and S_34_, are lower than 12 dB. Here, *S*_MN_ represents the ratio of the energy received from Port M to the energy fed through port N when energy is fed through port N. Figure 7b shows the mutual coupling between the vertically polarized feeding ports #5–#8. It is obvious that the isolation between adjacent ports is lower than 13 dB. Therefore, when designing the feeding networks for dual polarizations, the active impedance match of each port is carried out with consideration for the mutual coupling. Finally, there is a port isolation higher than 33 dB between dual polarizations in the desired band.

**Figure 8:**
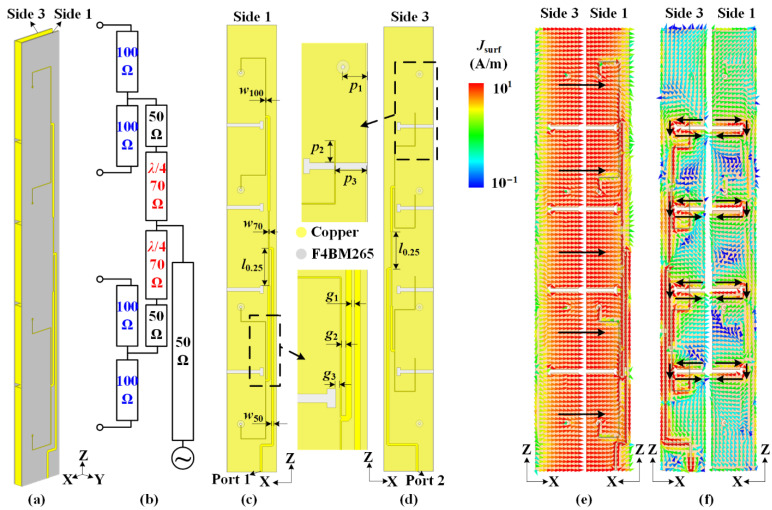
Design of feeding networks in the proposed antenna. (**a**) Three-dimensional perspective view of the proposed antenna. (**b**) Topological diagram of feeding networks for dual polarizations. Structures of feeding network for (**c**) horizontal polarization and (**d**) vertical polarization. Orthogonal current distributions on the cavity surface for high port isolation. Surface current distributions for (**e**) horizontal polarization and (**f**) vertical polarization.

As shown in Figure 8a, two feeding networks are fabricated on the substrates and located on Sides 1 and 3 of the cavity. Figure 8b shows the topological diagram of two feeding networks for each polarization. Due to the requirement for compact size, two quarter-wavelength impedance converters are adopted. The width of the 100 Ω-microstrip is *w*_100_ accepted for the printed circuit board (PCB) process. The feeding networks for two polarizations have a similar design strategy. The detailed views of the two feeding networks are shown in Figure 8c,d, respectively. To achieve a compact size, the folded slots are adjusted into a T-shape. By tuning the position *p*_1_ of the probe, the 100 Ω-microstrip causes the impedance to match horizontal polarization. For the same purpose, the impedance for vertical polarization is matched by tuning the values of *p*_2_ and *p*_3_. The detailed dimensions are as follows: *w*_50_ = 1.33 mm, *w*_70_ = 0.73 mm, *w*_100_ = 0.34 mm, *l*_0.25_ = 21 mm, *p*_1_ = 7.8 mm, *p*_2_ = 6.5 mm, *p*_3_ = 10.16 mm, *g*_1_ = 1 mm, *g*_2_ = 1 mm, and *g*_3_ = 1 mm.

It is a key issue that the orthogonally polarized antennas achieve a high port isolation. To verify this in the proposed antenna, the surface current distributions of the dual polarizations in the expanded view are depicted in Figure 8e,f. When the cavity is fed through Port 1 for horizontal polarization, as shown in Figure 8e, the total vector surface currents (black dash arrows) are in-phase on the opposite edges of the carved slots. When the group of folded slots are fed through Port 2 for vertical polarization, as shown in Figure 8f, it is observed that the surface currents are concentrated on the edges of the slots. Moreover, the vector surface currents are out-of-phase on the opposite edges of the slots. In algebraic terms, the current distributions of the horizontal and vertical polarizations are orthogonal in total. Therefore, a high port isolation is achieved within a compact ruler-like profile.

**Figure 9:**
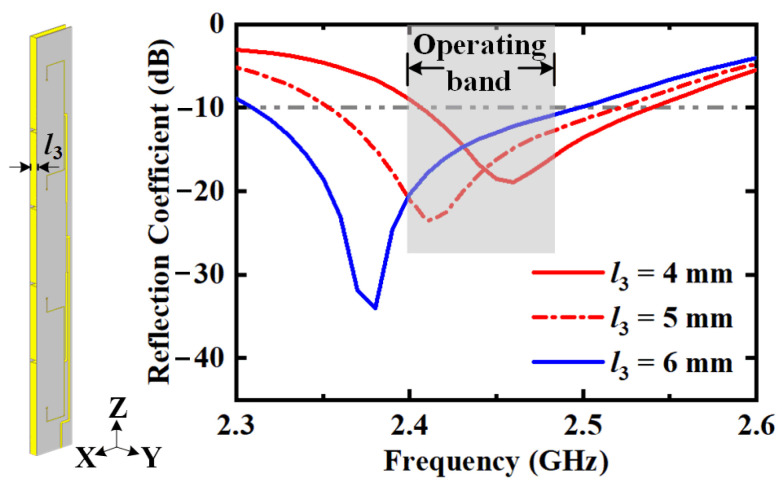
Reflection coefficients for horizontal polarization with different values of cavity thickness *l*_3_.

The operating bandwidth of horizontal polarization can be enhanced by increasing the cavity thickness *l*_3_. The reflection coefficients with different values of the cavity thickness *l*_3_ are depicted in Figure 9. As the cavity is thickened, the area of the radiating aperture becomes larger, reducing the quality factor of the cavity and broadening the bandwidth. Considering the trade-off between the volume of the antenna and the bandwidth of horizontal polarization, the value of cavity thickness is chosen as *l*_3_ = 5 mm in the proposed design. Similarly, the vertically polarized bandwidth can be enhanced by increasing the radiating aperture area, i.e., the slot width.

## 3. Fabrication and Measurement

**Figure 10:**
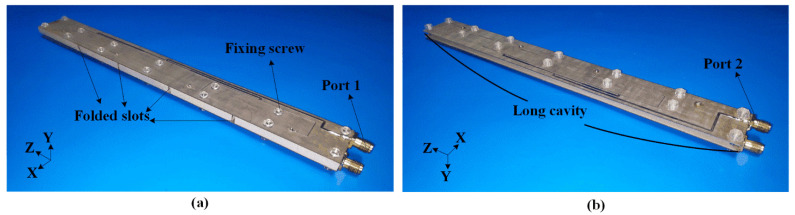
Photographs of the fabricated HGODP slot-cavity antenna. (**a**) Front view and (**b**) rear view. Feeding networks are fixed on the cavity with fourteen screws.

Figure 10 presents photographs of the fabricated prototype. The proposed HGODP antenna comprises a copper cavity with a thickness of 0.5 mm and two feeding networks on the substrates F4BM265 (*ε*_r_ = 2.65 and tan *δ* = 0.002). To connect these structures as a sandwich, several fixing screws are arranged in the proper place.

**Figure 11:**
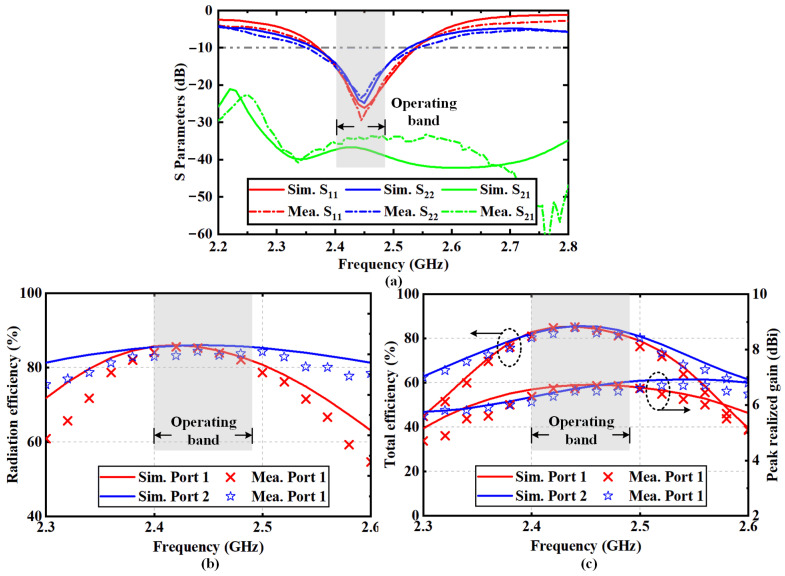
Simulated and measured results of the proposed antenna. (**a**) S-parameters, (**b**) radiation efficiencies and (**c**) peak realized gains and total efficiencies.

The measured and simulated *S*-parameters of the proposed antenna are illustrated in Figure 11a. The −10-dB impedance bandwidth is 2.37~2.54 GHz. The measured result of the port isolation is higher than 33 dB, indicating that a high isolation is achieved in the compact ruler-like structure. The discrepancies between the measurement and simulation are mainly attributed to fabrication errors. Figure 11b shows that the radiation efficiency of the proposed antenna is higher than 82% for each polarization. Figure 11c gives the peak realized gains and total antenna efficiencies. The proposed HGODP antenna achieves total efficiencies higher than 83% and 84% for the dual polarizations. On the other hand, peak realized gains of 6.7 dBi and 6.5 dBi are realized in the operating frequency band for the horizontal and vertical polarizations, respectively.

**Figure 12:**
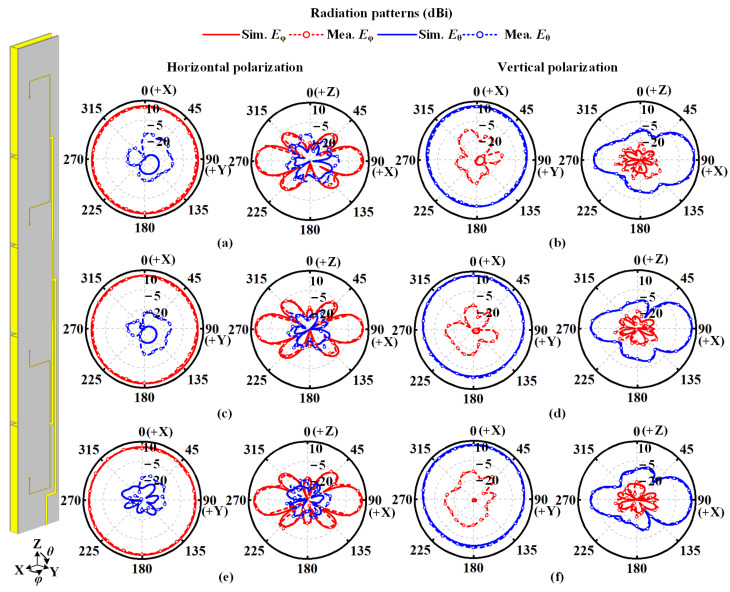
Measured and simulated radiation patterns for horizontal polarization at (**a**) 2.40, (**c**) 2.44, (**e**) 2.48 GHz, and vertical polarization at (**b**) 2.40, (**d**) 2.44, (**f**) 2.48 GHz.

Figure 12 illustrates the radiation patterns of the proposed antenna in the operating frequency band in simulation and measurement. As seen, the measured co-polarized gain variation of 2.2 dB is achieved for horizontal polarization, and the cross-polarization level is lower than −15 dB. For vertically polarized radiation, the proposed antenna realizes a co-polarized gain variation of 5.9 dB and cross-polarization of −14 dB in measurement. The measured radiation patterns in the XOZ plane also agree with the simulated ones. In addition, the measured and simulated radiation patterns at 2.40 and 2.48 GHz indicate that stable omnidirectional radiation are obtained in the operating frequency band.

Table 1 presents a comparison compared with the other literature in terms of the total volume, realized gain, port isolation, and operating frequency band. Compared with other HGODP antenna designs, the proposed antenna is designed within an extremely compact ruler-like structure with a thickness of only 0.04*λ*_0_. Besides, the proposed antenna provides a high port isolation of 33 dB, indicating that the sink nodes within the proposed antenna have the ability to receive orthogonally polarized signals from different sensor nodes in wireless sensor networks.

## 4. Conclusions

In this paper, a compact HGODP antenna is proposed within a slot-cavity structure by combining two high-gain omnidirectional antennas for orthogonal polarizations. A long cavity is designed and excited for a high aperture efficiency and horizontally polarized radiation. For vertical polarization, a group of slots is directly etched onto the cavity sidewalls. Due to the orthogonal operating modes of the dual polarizations, the proposed antenna achieves a high port isolation of more than 33 dB. The result also shows omnidirectional radiation is achieved for both dual polarizations. The proposed compact antenna has the advantages of high gain, omnidirectional radiation, and high isolation, exhibiting valuable potential for sink nodes in wireless sensor networks.

## Figures and Tables

**Table 1:** Comparisons of the proposed antenna with the other HGODP antenna in the literatures.

Ref.	Volume (*λ*_0_^3^)	Realized Gain for HP/VP (dBi)	Isolation (dB)	Band (GHz)
[16]	8.4 × 0.76 × 0.76	9.4/9.4	26	1.62~2.77
[17]	0.25 × π × 1.47^2^	5.0/5.4	20	1.71~2.69
[19]	0.23 × π × 0.34^2^	5.4/6.9	34	1.70~3.90
[21]	0.664 × 0.088 × 0.088	1.19/3.17	33.5	2.40~2.48
[23]	0.90 × 0.77 × 0.77	2.6/6.6	35	1.80~2.81
This work	2 × 0.23 × 0.04	6.7/6.5	33	2.37~2.54
